# Genetic diversity of *Plasmodium falciparum* isolates from concurrent malaria and arbovirus co-infections in Kedougou, southeastern Senegal

**DOI:** 10.1186/s12936-016-1208-7

**Published:** 2016-03-11

**Authors:** Makhtar Niang, Cheikh Loucoubar, Abdourahmane Sow, Moussa Moise Diagne, Oumar Faye, Ousmane Faye, Mawlouth Diallo, Aissatou Toure-Balde, Amadou A. Sall

**Affiliations:** Immunology Unit, Institut Pasteur Dakar, 36 Avenue Pasteur, 220 Dakar, Senegal; Arbovirus and Viral Hemorrhagic Fevers Unit, Institut Pasteur Dakar, 36 Avenue Pasteur, 220 Dakar, Senegal; Medical Entomology Unit, Institut Pasteur Dakar, 36 Avenue Pasteur, 220 Dakar, Senegal

**Keywords:** Malaria, Arbovirus, Genetic diversity, Multiplicity of infection

## Abstract

**Background:**

Concurrent malaria and arbovirus infections are common and represent an important public health concern in regions where both diseases are endemic. The present study investigates the genetic diversity and complexity of *Plasmodium falciparum* infection in concurrent malaria-arbovirus infections in Kedougou region, southeastern Senegal.

**Methods:**

Parasite DNA was extracted from 60 to 27 sera samples collected from *P. falciparum* isolates of malaria and concurrent malaria-arbovirus infected patients, respectively, and followed by PCR-genotyping targeting the *msp*-*1* (block2) and *msp*-*2* (block3) allelic families.

**Results:**

The mean number of genotype per allelic family was comparable between the two groups. K1 was the predominant *msp*-*1* allelic type both in malaria (94.91 %) and arbovirus-malaria (92.59 %) groups, whereas IC/3D7 was the most prevalent *msp*-*2* allelic type in malaria (94.91 %) and arbovirus-malaria (96.29 %) groups. Frequencies of *msp*-*1* and *msp*-*2* allelic types were statistically comparable between the two groups (Fisher exact test, P > 0.05) and were not associated with age. FC27 was strikingly the least prevalent in both groups and was absent in children under 5 years of age. The proportions of *P. falciparum* isolates from malaria-infected patients carrying the three *msp*-*1* allelic types (67.44 %) or the two *msp*-*2* allelic types (76.47 %) were significantly higher than those from arbovirus-malaria co-infected patients (Exact binomial test, P < 0.05). The multiplicities of infection (MOI) were low and comparable for *msp*-*1* (1.19 vs 1.22) and *msp*-*2* (1.11 vs 1.10), respectively between malaria and arbovirus-malaria groups.

**Conclusion:**

The study showed no difference in the genetic diversity between *P. falciparum* isolates from malaria and concurrent malaria-arbovirus infected patients in Kedougou. The MOI was low despite intense malaria transmission in Kedougou. The overall results suggest a limited or no influence of arbovirus infections on *P. falciparum* diversity and complexity of malaria infection.

## Background

Malaria and arboviruses infections are common arthropod-borne diseases in Africa and represent major public health problems, as both diseases have been independently associated with significant morbidity and mortality [1, 2]. Concurrent malaria and arbovirus infections have been reported previously [3–5], but its burden, clinical significance and public health impact remain elusive mostly due to the lack of diagnostics capacities for arboviruses or the low sensitivity of malaria detection methods in countries where the two diseases are prevalent or endemic [6–8]. A recent study reported frequent concurrent malaria parasite and arbovirus infections in acute febrile patients in Kedougou region (southeastern Senegal) strongly associated with high fever (≥40 °C) compared to malaria or arbovirus infection alone [9]. To date, relatively little is known with regards to the nature of precise interactions during concurrent malaria-arbovirus infections.

While the interactions of malaria with parasitic infections, such as helminths, or viral infections, such as HIV, have been extensively documented [10–12], relatively little is known with regards to malaria and arboviruses co-infections. For example, investigations of the nature of interactions between helminths and malaria led to contradictory results probably as the result of the complex nature of the immune response to malaria parasites, due to the antigenic diversity of Plasmodium stages, or the altered immune responses resulting from co-infections [13]. The latter altered immune responses have been associated with greater complexity of malaria infection and parasite genetic diversity, one of the limitations to the development of an effective vaccine against *Plasmodium falciparum* malaria [14, 15].

Since malaria and arboviral infections often coincide geographically in the same tropical regions, the question arises whether arboviral infections modulate the immune responses towards the malaria parasite and affect the course and the complexity of malaria infection. A profound effect of arboviral infections on the immune system might be expected to influence the immune response against malaria parasites, thus affecting the course of infection and contributing to the complexity of the malaria disease.

In malaria endemic regions, studies have demonstrated that individuals can be simultaneously infected with multiple *P. falciparum* populations that are genetically distinct [16–19]. Such multiple infections are believed to play an important role in the development of strain-specific immunity and have been associated with increased risk of treatment failure [20–22]. The *P. falciparum* merozoite surface proteins (*msp*-*1* and *msp*-*2*) have been widely used as markers to investigate the genetic diversity, multiplicity of infection, level of malaria transmission, as well as relationship with immunity against malaria [23, 24].

As no study has been reported so far on the extent of the genetic diversity of *P. falciparum* isolates in concurrent malarial infection, this study investigated the genetic diversity of *P. falciparum* isolates from patients infected only with malaria parasites or co-infected with arboviruses and malaria parasites, in Kedougou, southeastern region of Senegal.

## Methods

### Population study

A total of 101 sera from patients that have visited healthcare facilities in the Kedougou region between July 2009 and July 2013 were withdrawn from a collection established as part of a project investigating dengue infections in Kedougou region and investigated in this study. Due to the similar clinical presentation between malaria and arboviral infection, malaria diagnostic was systematically conducted to differentiate arboviral infection to malaria. During inclusion, all patients presented with acute febrile illness (AFI) which was defined as “any patient older than 1 year with a fever (temperature >38 °C) lasting for less than 2 weeks, exhibiting two or more of the following symptoms: headache, myalgia, eye pain, arthralgia, cough, nausea/vomiting, diarrhoea, jaundice, bleeding and neurological signs”. Clinical manifestations and socio-demographic data were recorded on a standardized interview form.

Study objectives, benefits and risks were explained in French or local languages to all participants before inclusion. Written informed consent was obtained from adults and parents or legal guardians of children. The study was examined and approved by the Senegalese National Health Research Ethical Committee.

### Malaria and arbovirus laboratory diagnostics

All 101 sera were systematically tested for *P. falciparum* malaria and arboviruses infections. Malaria diagnostic was initially performed by microscopic examination of Giemsa-stained thick blood smears and/or a rapid diagnostic test (Malaria Antigen P.f, Standard diagnostics, INC. Ingbert, Germany) while IgM antibodies capture ELISA, real-time RT-PCR and virus isolation methods [25, 26] were used for detection of arboviruses.

Consequently, “arbovirus confirmed case” was defined as “any AFI tested positive for any method used for detection of IgM and/or genome of ZIKV, DENV, CHIKV, RVFV, CCHFV, WNV or YFV” while malaria “confirmed case” was considered for “any AFI tested positive by RDT and/or microscopy”. Concurrent infection was defined as “any AFI confirmed for any arbovirus infection and malaria”.

### Molecular detection of *Plasmodium falciparum*

The detection of Plasmodium DNA in frozen serum samples has been reported earlier [27, 28]. Genomic DNA isolation of Plasmodium parasites was performed using QIAamp DNA Blood Mini Kit (Qiagen, Hilden, Germany) according to manufacturer’s instructions with minor modifications. Hundred microlitres (100 µl) of serum samples were used for extraction instead of 200 µl and final elution of isolated DNA was performed in 50 µl volume. DNA was stored at −20 °C until further analysis. DNA extracted from 100 µl of heparinized whole blood samples from known smear-positive *P. falciparum* malaria patient was used as positive control.

Qualitative detection of *P. falciparum* parasite DNA was based on nested PCR amplification with primers targeting the *Plasmodium* spp 18S small subunit ribosomal RNA gene as described by Snounou and others [29]. The primary PCR amplification was performed with Plasmodium genus specific rPLU5 and rPLU6 primers pairs [30, 31] and 2 µl of extracted DNA in a total volume of 25 µl using the GoTaq Green Master Mix protocol (Catalog no M7113, Promega). The nested reaction was performed for the specific detection of *P. falciparum* using previously described primers pairs rFAL1 and rFAL2 [29, 31] in a total volume of 25 µl containing 1 µl of the primary PCR product. Nested PCR results were scored as categorical variables (presence vs absence of amplification). PCR cycling reactions and amplification conditions were as described by Snounou and Singh [29].

### Molecular genotyping of *Plasmodium falciparum* isolates

*Plasmodium falciparum* isolates from malaria and concurrent arbovirus-malaria infected patients were analysed by amplification of the two highly polymorphic regions of *msp*-*1* (block2) and *msp*-*2* (block 3) using nested PCR as described previously [32, 33]. Sequences of primers sets used for detecting the three families of *msp*-*1* (K1, MAD20 and RO33) and two families of *msp*-*2* (FC27 and 3D7) are reported elsewhere [33–35]. An initial amplification of the outer regions of the two genes was followed by a nested-PCR with allelic family specific primers pairs. In the first round reaction, 2 µl of genomic DNA was added as a template while in the nested reaction, 1 µl of the outer PCR product was used as template. Amplification products were separated by electrophoresis on 1.5 % agarose gel, and the fragments were visualized under UV light with ethidium bromide. The sizes were determined using a molecular weight marker.

The frequency of each *msp* family was calculated as the percentage of samples containing at least one allele from that family. The mean multiplicity of infection (MOI) or number of genotypes per infection [24, 36] was calculated by dividing the total number of fragments detected in *msp*-*1* or *msp*-*2* by the number of samples positive for the same marker.

### Statistical analysis

Data were analysed using R statistical software version 3.1.1 (2014-07-10) [37]. We performed Kruskal–Wallis and Fisher’s exact tests to compare distribution of quantitative and qualitative variables respectively between subgroups. When pair-wise comparisons were performed between more than two groups, P values corrected for multiple testing by the Bonferroni method were performed. Statistical significance was considered when P values were less than or equal to 0.05.

## Results

### Characteristics of the study population

**Fig. 1:**
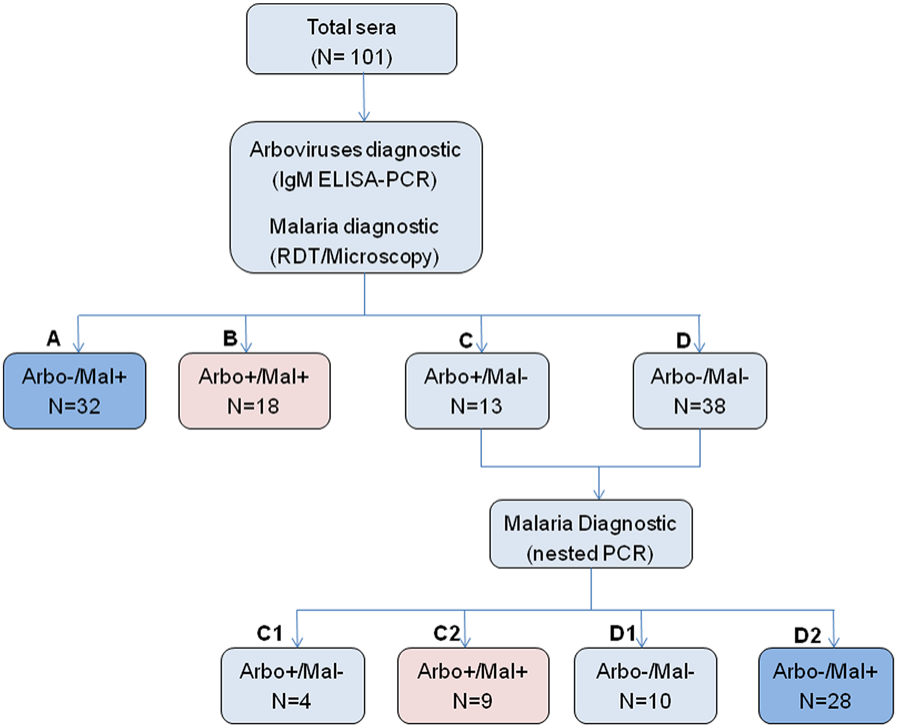
Flow chart describing the selection of the malaria and malaria-arboviruses infected samples

Sera from 101 patients aged between one and 59 years were retrieved from the sera bank and investigated in this study for *P. falciparum* and arboviruses infections. Among them, 32 were uniquely positive for *P. falciparum* based on RDT and/or microscopy examination (Fig. 1, branch A), 18 were concurrently positive for *P. falciparum* and arboviral infections (Fig. 1, branch B), 13 were uniquely infected by arboviruses (Fig. 1, branch C), while 38 were negative for both arboviral and malaria infections (Fig. 1, branch D). Due to the limited sensitivity of RDT and microscopy to detect low-density parasitaemia, nested PCR was performed on the 51 malaria negative samples (C + D) for further detection of malaria parasites. This revealed 37 additional *P. falciparum* positive samples, of which 9 were co-infected with an arbovirus (Fig. 1, branch C2) while 28 were uniquely infected by malaria parasite (Fig. 1, branch D2). This raises the number of unique *P. falciparum* malaria and concurrent malaria-arboviruses infections to 60 (A + D2) and 27 (B + C2), respectively (Fig. 1).

The sex ratios M/F were 1.40 (35/25) and 1.07 (14/13) for the malaria and arbovirus-malaria groups respectively (Table 1). The mean age was 20.13 (1–60) and 22.07 (2–50) years respectively for the malaria and arbovirus-malaria groups (Table 1). The malaria and arbovirus-malaria groups were comparable in term of age (Kruskal–Wallis *P* value = 0.59) and sex (Fisher’s exact P value = 0.64).

**Table 1:** Baseline characteristics of the study population

	Malaria (N = 60)	Arbovirus-malaria (N = 27)	OR (95 % CI)	P value
	N (%)	N (%)		
Age (year)				
Mean (SD)	20.13 (14.2)	22.07 (15.4)		0.59
(Range)	(1–60)	(2–50)		
Sex				
Male	35 (58.3)	14 (51.8)	–	
Female	25 (41.7)	13 (48.2)	1.29 (0.47–3.57)	0.64
Ratio M/F	1.40	1.07		
Age groups (years)				
≤5	8 (14.94)	5 (18.51)	–	
(5–15)	17 (26.43)	6 (22.22)	0.57 (0.11–3.15)	0.68
>15	35 (58.62)	16 (59.25)	0.74 (0.18–3.33)	0.86

### Genetic diversity of *Plasmodium falciparum* isolates

Parasites DNA from all 60 *P. falciparum*-infected and 27 concurrent *P. falciparum*-arbovirus co-infected samples were successfully genotyped. All three families of *msp*-*1* (K1, MAD20 and RO33) and two families of *msp*-*2* (IC/3D7 and FC27) were detected in *P. falciparum* isolates from both malaria and arbovirus-malaria co-infected patients.

In the malaria group, 40.46 % (70/173) of the overall detected *msp*-*1* genotypes belonged to MAD20 allelic family while K1 and RO33 allelic families represented respectively 32.94 % (57/173) and 26.58 % (46/173) of the total *msp*-*1* genotypes (Table 2). With respect to arbovirus-malaria co-infected group, the MAD20 family was also the predominant *msp*-*1* genotype and represented 40 % (34/85) of the overall detected *msp*-*1* genotypes whereas K1 and RO33 allelic families represented 30.58 % (26/85) and 29.41 % (25/85), respectively (Table 2).

**Table 2:** Total and mean number of genotype per allelic family

Total (mean)	*msp*-*1*				*msp*-*2*		
	K1	MAD20	RO33	Total	IC/3D7	FC27	Total
Malaria	57 (0.96)	70 (1.18)	46 (0.77)	173	60 (1.01)	17 (0.28)	77
Arbovirus-malaria	26 (0.96)	34 (1.21)	25 (0.89)	85	30 (1.07)	4 (0.14)	34

The IC/3D7 allelic type was the predominant *msp*-*2* genotype in both malaria and arbovirus-malaria groups with respectively 77.92 % (60/77) and 88.23 % (30/34) of the total detected *msp*-*2* genotypes. FC27 allelic type represented only 22.07 % (17/77) and 11.76 % (4/34) of *msp*-*2* genotypes in malaria and arbovirus-malaria groups, respectively (Table 2).

For a given *msp*-*1* or *msp*-*2* allelic family, the total number of genotypes was higher in the malaria group than in the arbovirus-malaria group. However, the mean number of genotype per allelic family was comparable between the two groups (0.96 vs 0.96 for K1, 1.18 vs 1.21 for MAD20, 0.77 vs 0.89 for RO33, 1.01 vs 1.07 for IC/3D7 and 0.28 vs 0.14 for FC27). This suggests that the elevated number of genotypes observed in the malaria group could be due to the higher number of samples from malaria-infected patients rather than a lower genetic diversity of *P. falciparum* isolates from concurrent arbovirus-malaria infected patients.

### Frequency of *msp*-*1* and *msp*-*2* allelic types in the study groups

**Fig. 2:**
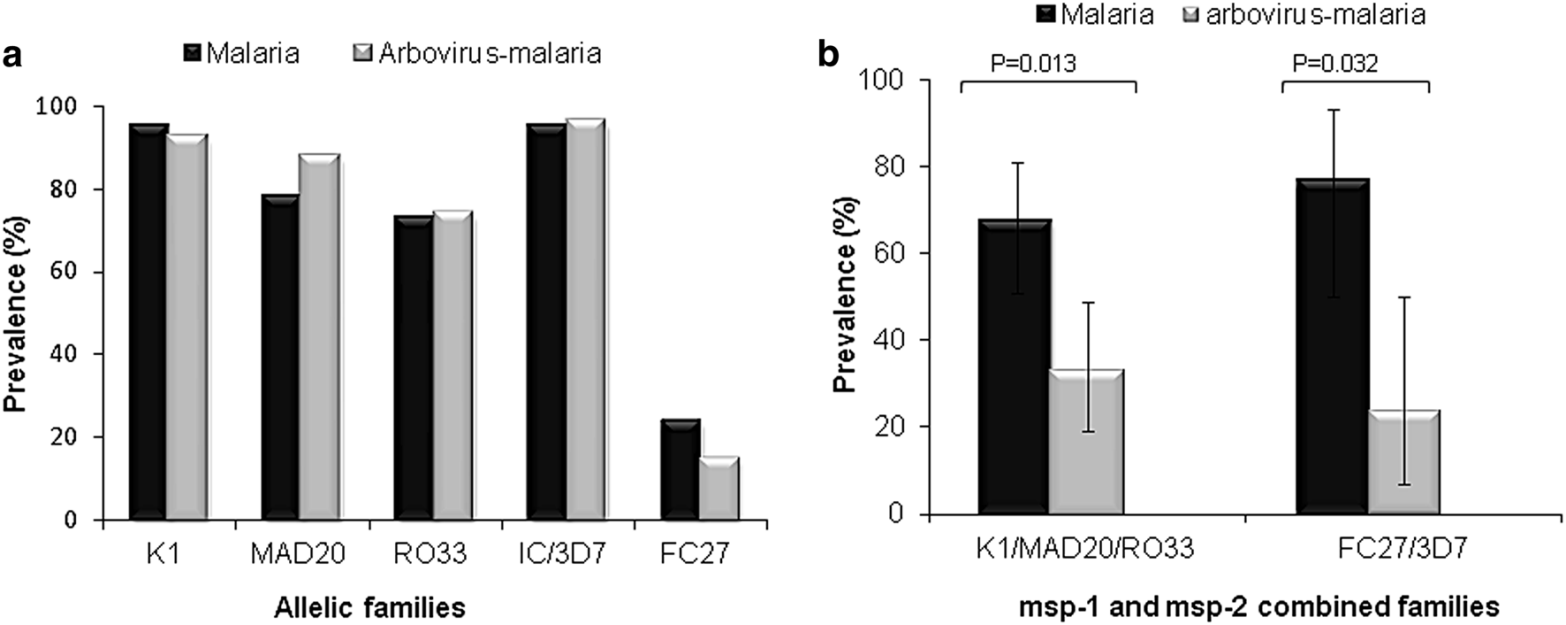
Frequency of individual (**a**) or combined (**b**) *msp-1* and *msp-2* allelic families of *Plasmodium falciparum* isolates from malaria and malaria-arbovirus infected patients

The frequency of K1, MAD20 and RO33 allelic families of *msp*-*1* and IC/3D7 and FC27 allelic families of *msp*-*2* were compared between malaria and arbovirus-malaria groups. In both groups, K1 and IC/3D7 families were respectively the predominant *msp*-*1* and *msp*-*2* allelic types (Fig. 2a). The frequencies of *msp*-*1* specific allelic types were statistically comparable between the malaria and arbovirus-malaria groups and were respectively 95 vs 92.59 % for K1 (P value = 0.64), 78.33 vs 81.48 % for MAD20 (P value = 0.99) and 73.33 vs 74.07 % for RO33 (P value = 0.99) (Fig. 2a).

Similar to *msp*-*1* allelic types, the frequencies of *msp*-*2* specific allelic types were also statistically comparable between *P. falciparum* isolates from malaria and arbovirus-malaria patients and were 93.33 vs 96.29 for IC/3D7 (P value = 0.99) and 23.33 vs 14.81 % for FC27 (P value = 0.57) (Fig. 2a). FC27 was strikingly the least prevalent allelic family in both groups (Fig. 2a).

The proportion of *P. falciparum* isolates carrying simultaneously the three *msp*-*1* allelic types and the two *msp*-*2* allelic types were also compared between the two groups and reported in Fig. 2b. The proportion of *P. falciparum* isolates from malaria-infected patients carrying the three *msp*-*1* allelic types (67.44 %) was significantly higher than that from arbovirus-malaria co-infected patients (32.56 %) (Exact binomial test, P = 0.013). A similar distribution was observed between *P. falciparum* isolates carrying the two *msp*-*2* allelic types from simple malaria (76.47 %) and arbovirus-malaria (23.53 %) cases (Exact binomial test, P = 0.032) (Fig. 2b).

### Distribution of *msp*-*1* and *msp*-*2* allelic families according to age groups

**Fig. 3:**
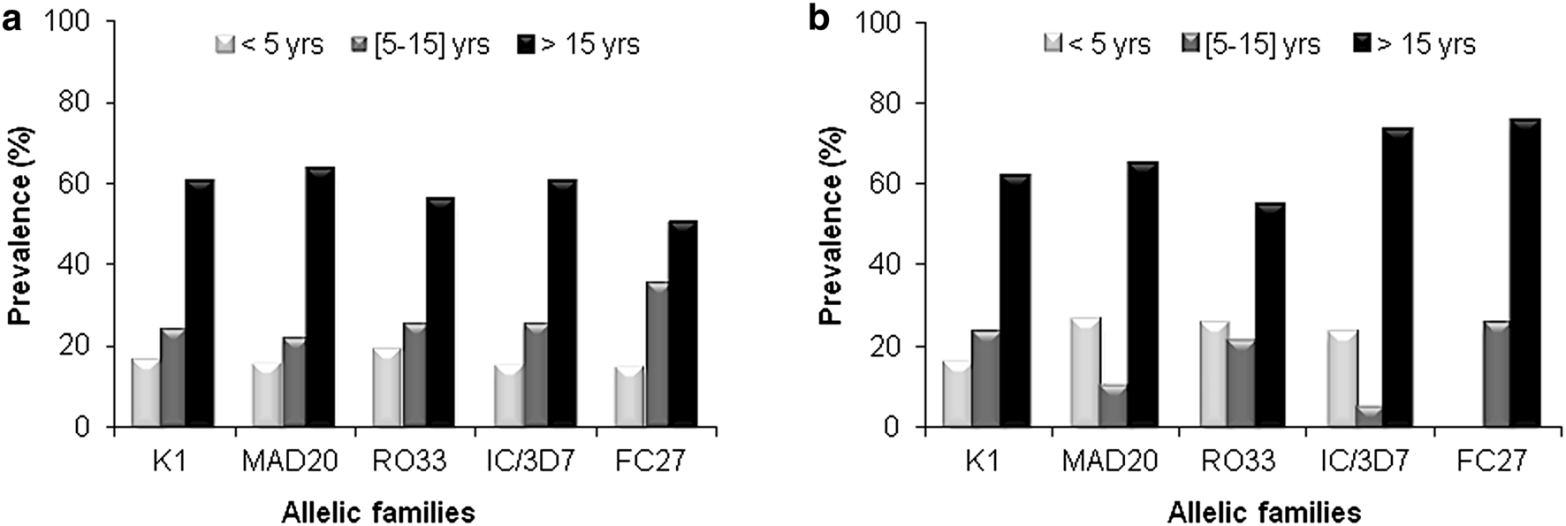
Distribution of *msp*-*1* and *msp*-*2* allelic families of *Plasmodium falciparum* isolates from malaria (**a**) and concurrent malaria and arbovirus (**b**) infected patients according to age groups

The frequency of specific *msp*-*1* and *msp*-*2* allelic families were categorized within three age groups: ≤5, (5–15) and >15 years (Table 1). For a given *msp*-*1* and *msp*-*2* allelic family, there was an age-dependant distribution of the frequency of allelic types from *P. falciparum* malaria-infected isolates with patients in the oldest age group (>15 years) showing the highest frequency (Fig. 3a). Frequencies of the three *msp*-*1* allelic types (K1, MAD20 and RO33) as well as the two *msp*-*2* allelic types (IC/3D7 and FC27) were comparable within age groups.

By contrast, the distribution of *msp*-*2* allelic types of *P. falciparum* isolates from arbovirus-malaria co-infected patients according to age groups showed a slightly different pattern (Fig. 2b) characterized by a lower frequency of MAD20, RO33 and IC/3D7 allelic famiilies in the (5–15 years) age group and an absence of FC27 allelic family in children below 5 years old (Fig. 3b). However, no significant difference was found for K1 (P = 0.89), MAD20 (P = 0.54), RO33 (P = 0.88), IC/3D7 (P = 0.89) and FC27 (P = 1.00) when comparing age-distribution of a given allelic family between malaria and arbovirus-malaria groups using Fisher exact test.

### Multiplicity of *Plasmodium falciparum* infection

**Fig. 4:**
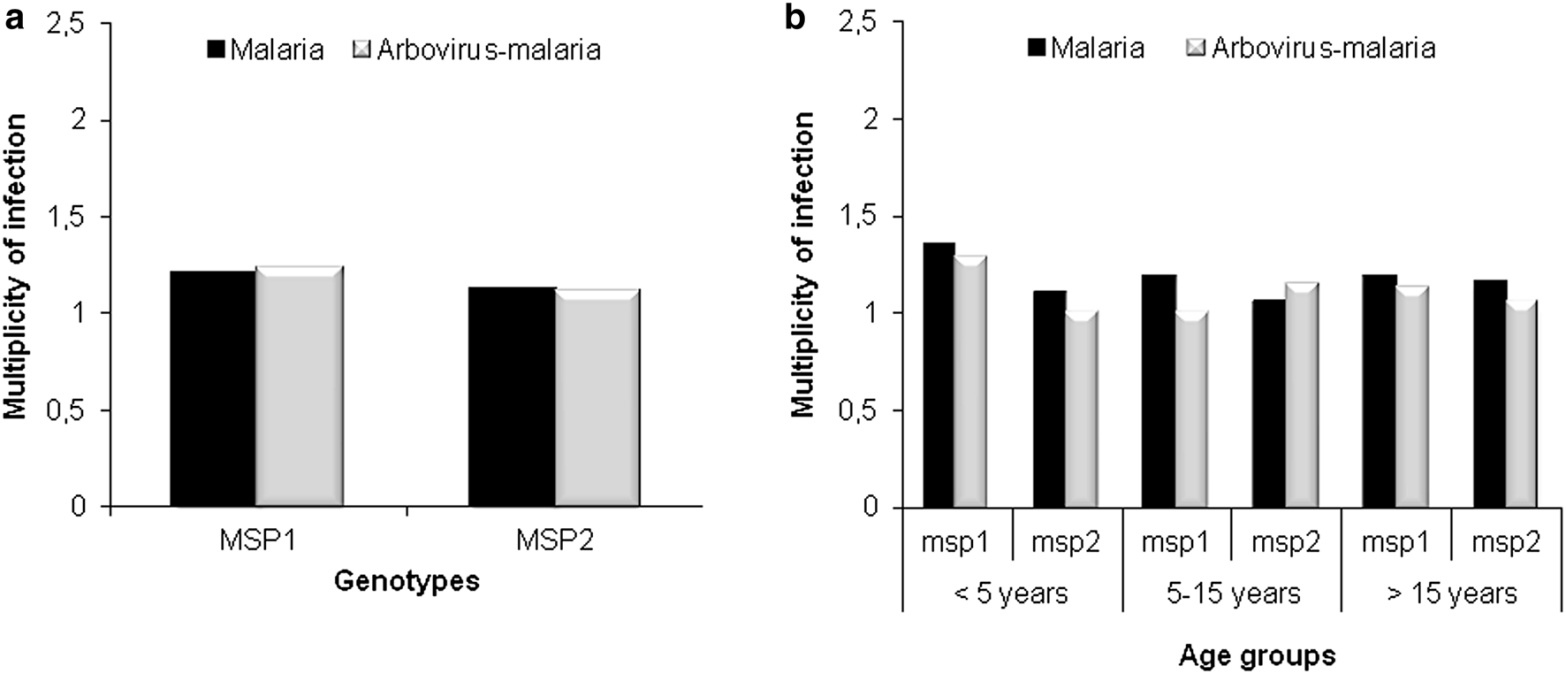
Distribution of *msp*-*1* and *msp*-*2* multiplicity of infection in malaria and arbovirus-malaria groups (**a**) and their distribution according to age groups (**b**)

The mean multiplicity of *P. falciparum* infection (MOI) was found to be 1.19 and 1.11 respectively for *msp*-*1* and *msp*-*2* in the malaria group (Fig. 4a). In the arbovirus-malaria group, the mean MOI was 1.22 and 1.1 for *msp*-*1* and *msp*-*2,* respectively (Fig. 4a). The distribution of *msp*-*1* and *msp*-*2* mulitiplicity of infection according to the three age groups showed no association with age (Fig. 4b). The difference in MOI for *msp*-*1* and *msp*-*2* between malaria and arbovirus-malaria groups as well between age groups was not statistically significant (P > 0.05).

## Discussion

In resources-limited settings, given the limited number of laboratories diagnosing arboviral infections along with malaria infections, reports on malaria and arbovirus co-infections are scarce and their prevalence underestimated [3–5, 9, 38]. PCR has been shown to be more reliable in detecting malaria parasite [6, 7, 39] as confirmed in the current study showing that not only PCR could detect malaria parasite in all microscopy-positive samples but also in samples found negative by microscopy [7, 40]. The study showed that microscopy-based diagnostic of *P. falciparum* highly underestimated malaria parasite carriage. Importantly, *Plasmodium* DNA was detected from frozen serum samples similar to previous reports [27, 28], providing the potential for retrospective diagnostic of active malaria infections in patients cohorts to determine potential interactions of malaria with others diseases.

In this study, the genetic diversity exhibited by *P. falciparum* isolates from malaria-infected patients and concurrent arboviruses-malaria co-infected patients was investigated in Kedougou region with an ultimate goal of determining whether co-infection with an arbovirus contributed to the overall genetic diversity of *P. falciparum* isolates and complexity of malaria infections. Both malaria and arbovirus-malaria groups were comparable in term of age, sex and percentage of patients within defined age groups.

The comparable mean number of genotype for each locus between malaria and arbovirus-malaria groups suggested that the elevated number of genotypes observed in the malaria group could be related to the higher number of samples rather than an extended genetic diversity of *P. falciparum* isolates from malaria-infected patients. In this study, the frequency of all *msp*-*1* allelic families (K1, MAD20 and RO33) and IC/3D7 allelic family of *msp*-*2* were high (>70 %) and comparable between the two study groups. The current parasite profile with the predominance of K1 and IC/3D7 allelic families was also reported in others studies [41–43]. The highly significant differences observed between the proportions of *P. falciparum* isolates carrying either the three *msp*-*1* allelic types or the two *msp*-*2* allelic types from malaria-infected patients compared to arbovirus-malaria co-infected patients, are probably uniquely due to elevated number of sample concerning simple infection compared to co-infection. The distribution of *msp*-*1* and *msp*-*2* families according to age groups was similar between malaria and arbovirus-malaria groups and no significant difference was found when comparing distribution of a given allelic family between the two groups despite the absence of FC27 family in younger children (<5 years).

The MOI reported in this study was not influenced by age similar to reports from others studies [44]. This contrasts with studies that have shown a greater MOI in adults [45, 46] as well as findings of decreased MOI with age [47, 48]. The lack of association between MOI and age could suggest that the MOI is not directly related to the period of acquisition of immunity. Thus, a study of the MOI and immunity against asexual stages of *P. falciparum* showed that samples with the highest MOI came from children with significantly lower antibody responses to specific antigens of the asexual parasite [43]. Malaria episodes with many clones would induce a diversity-driven delayed acquired immunity with consequent limited ability to control malaria infection. The low allelic diversity together with the high frequency of the circulating allelic families increases the chance of the re-infection with the same allele.

Although the sera investigated in this study spanned a 5-year period and originated from different health facilities, possibly affecting the MOI as previously reported [49], the spatial and temporal dependencies of the different genotypes were not analyzed in the present study. The frequency and distribution of *msp*-*1* and *msp*-*2* allelic families with respect to sampling period and health facilities/villages of origin are being investigated in a more extensive study using 160 *P. falciparum* isolates from Kedougou (Niang M, personal communication).

## Conclusion

This study provides the first information on the genetic diversity of *P. falciparum* isolates and complexity of infection in concurrent arbovirus-malaria infections. The study revealed no influence of arboviral infection on the genetic diversity of *P. falciparum* isolates as well as complexity of *P. falciparum* malaria infections in Kedougou. The study did not find any *msp*-*1* and/or *msp*-*2* allelic type frequency, MOI or distribution of a given allelic type or MOI with age groups that would suggest a role of arboviruses infections on the genetic diversity of *P. falciparum* isolates and complexity of malaria infection in Kedougou. Other studies that examine *P. falciparum* population structure and, taking into account the nature of anti-malarial immune responses are needed to better document the influence of arboviruses infections on *P. falciparum* genetic diversity and complexity of malaria infection.

## Abbreviations

YFV: yellow fever virus
ZIKV: zika virus
CHIKV: chikungunya virus
DENV: dengue virus
CCHFV: Crimea Congo hemorrhagic fever virus
WNV: West Nile virus
RDT: rapid diagnostic test
AFI: acute febrile illnesses
ELISA: enzyme-linked immuno assay
